# Symptomatic Predictors for 2009 Influenza A Virus (H1N1) Infection with an Emphasis for Patients with a Negative Rapid Diagnostic Test

**DOI:** 10.1371/journal.pone.0028102

**Published:** 2011-12-02

**Authors:** Chen-Yen Kuo, Yhu-Chering Huang, Chung-Guei Huang, Kuo-Chien Tsao, Tzou-Yien Lin

**Affiliations:** 1 Department of Pediatrics, Chang Gung Memorial Hospital, College of Medicine, Chang Gung University, Taoyuan, Taiwan; 2 Graduate Institute of Clinical Medical Sciences, College of Medicine, Chang Gung University, Taoyuan, Taiwan; 3 Department of Laboratory Medicine, Chang Gung Memorial Hospital, Taoyuan, Taiwan; University of Hong Kong, Hong Kong

## Abstract

**Background:**

The clinical diagnosis of influenza is difficult because it shares nonspecific symptoms with a variety of diseases. Emergency departments and clinics were overwhelmed by a surge of anxious patients during the 2009 influenza A virus (H1N1) outbreak. Our objective was to identify symptomatic predictors of influenza virus infection for patients with a negative rapid diagnostic test.

**Methodology/Principal Findings:**

We conducted a retrospective review of 805 patients who presented at Chang Gung Memorial Hospital, from August 1, 2009, to September 30, 2009. Respiratory specimens from these patients were subjected to rapid influenza tests and reverse-transcription polymerase chain reactions. In total, 36% of 308 children and 23% of 497 adults were positive for 2009 influenza A virus (H1N1) infection by polymerase chain reaction or virus culture. For pediatric patients, sore throat and influenza-like illness significantly increased the odds of having 2009 influenza A virus (H1N1) infection, by more than 3-fold (95% confidence interval (CI): 1.9–7.3) and 7-fold (95% CI: 4.00–14.2), respectively. For adult patients, cough and constitutional symptoms increased the odds of having 2009 influenza A virus (H1N1) by greater than 5-fold (95% CI: 3.1–10.2) and 3-fold (95% CI: 2.1–6.7), respectively. The negative likelihood ratio of the combination of fever and cough was 0.096 (95% CI: 0.01–0.69) for children with negative results of rapid influenza diagnostic tests.

**Conclusion/Significance:**

In influenza epidemic settings, clinicians should be aware that rapid influenza diagnostic tests are relatively insensitive for the diagnosis of influenza virus infection. For patients with negative rapid influenza diagnostic tests, those lacking fever and cough have a low probability of influenza virus infection. The management strategy should be made individually and depend on the severity of illness.

## Introduction

Influenza virus infection is a major public-health problem that affects 5–15% of the global population annually [1]. Given its propensity for antigenic drifts and shifts, influenza has the capacity to cause annual epidemics and occasional pandemics [2]. Appropriate and prompt diagnosis and therapy affect society as well as individual patients, because local outbreaks may be detected and control measures initiated [3]. However, influenza is difficult to diagnose clinically because the symptoms are largely nonspecific and a variety of diseases can cause similar symptoms. A symptom complex for influenza-like illness (ILI) has been used as a predictive tool for the diagnosis of influenza infection at the primary-care level, especially in influenza epidemic contexts. However, the sensitivity and positive predictive values of such tools vary widely and depend on the prevalence of influenza, the population tested, and the co-circulation of other respiratory viruses in the community [4].

Several laboratory assays are available for the diagnosis of influenza, including viral cultures, serology, rapid diagnostic (antigen) testing, reverse-transcription polymerase chain reaction (RT-PCR), and immunofluorescence assays [5]–[9]. Rapid antigen detection assays, including chromatographic immunoassays, are used widely because they are relatively easy to handle, less costly, and provide test results in less than 15 min [8], [10]–[12]. The sensitivity and specificity of any test for influenza can vary depending on the laboratory performing the test, test and specimen types, specimen quality, and the timing of specimen collection in relation to illness onset.

A pandemic outbreak of a novel strain of influenza A (H1N1) virus, first identified in Mexico, occurred from March 2009 onward [13]–[15]. News of the pandemic led to a heightened awareness of the consequences of influenza. Apprehension skyrocketed in the general population and among healthcare providers, causing substantial increases in the number of patient visits to hospital emergency departments.

In Taiwan, 2009 influenza A (H1N1) virus became circulating in the communities since July 2009. The Centers for Disease Control of Taiwan (CDC- Taiwan) published the “Clinical Treatment Guidelines for Influenza A (H1N1)” on August 17, 2009. According to the guidelines, oseltamivir was suggested to be prescribed to patients who had influenza-like illness and also had either a positive rapid influenza diagnostic test result, complicated influenza, at-risk conditions for complications defined by the World Health Organization (WHO), or hazardous signs defined by CDC-Taiwan, and the medication would be provided by CDC-Taiwan. But, the costs of rapid influenza diagnostic tests were needed to be paid by patients themselves. Emergency departments and clinics became overwhelmed by a surge of anxious patients who presented with respiratory illnesses of varying severity. The increased diagnostic testing needs caused increases in the workload of hospital personnel and testing demands on laboratory resources.

To reduce unnecessary diagnostic testing and demands on laboratory resources in the context of influenza epidemics, we undertook a comparative study to identify symptomatic predictors of influenza virus infection, especially for patients with a negative rapid diagnostic test.

## Methods

### Ethics Statement

This study was approved by the institutional review board of Chang Gung Memorial Hospital. Informed consent was omitted because the data were analyzed anonymously.

### Definition of influenza virus infection

Influenza virus infection was defined as a positive result of reverse-transcription polymerase chain reaction (RT-PCR) or virus culture for influenza virus. Influenza-like illness was defined according to the criteria proposed by CDC-Taiwan: the presence of fever plus at least one upper-respiratory symptom (cough, sore throat, rhinorrhea) and one constitutional symptom (headache, malaise, myalgia) [16].

### Study population

**Figure 1:**
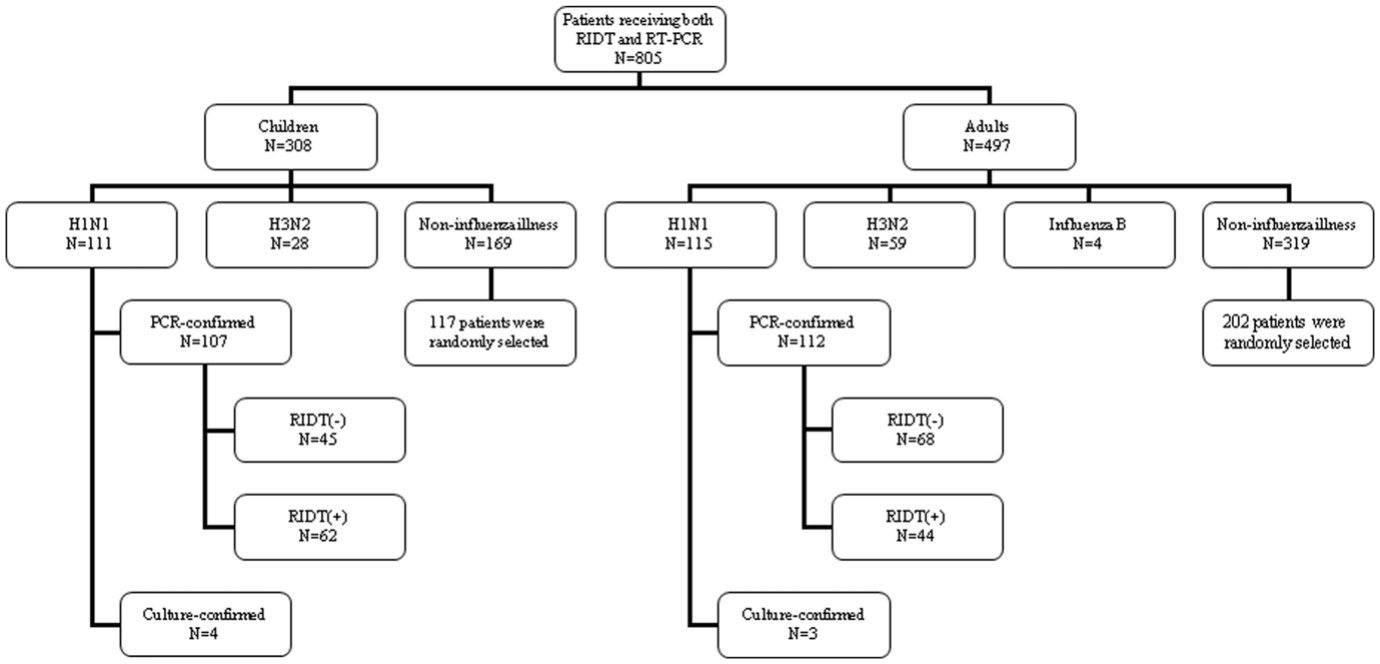
Flow chart of patient inclusion, including 226 cases of 2009 influenza A virus (H1N1) infection and 319 cases of non-influenza controls. RIDT, rapid influenza diagnostic test; RT-PCR, reverse transcriptase polymerase chain reaction.

Between August 1 and September 30, 2009, at Chang Gung Memorial Hospital, Taoyuan, Taiwan, a total of 805 patients (308 children, 497 adults) with suspected influenza virus infection receiving both rapid influenza diagnostic test and RT-PCR for influenza virus were enrolled in this study. Virus isolation and identification was further performed for 132 of these patients. The specimens were obtained either from nasopharyngeal or throat swabs. The decision to collect a nasopharyngeal or throat swab sample was made at the discretion of the individual treating physician. These specimens were transported to the virology laboratory and processed immediately. Overall, 317 (39%) patients had influenza virus infection: 226 patients (111 children, 115 adults) had 2009 influenza A virus (H1N1) infection, 87 patients (28 children, 59 adults) had influenza A (H3N2) virus infection, and four adult patients had influenza B virus infection. In this study, we focused on the patients with 2009 influenza A virus (H1N1) infection in terms of having a homogeneous group of patients to study. 117 of 169 children and 202 of 319 adults with negative results for influenza virus were randomly selected as controls, with a ratio of 2 to 3 by an age interval of 10 years. Figure 1 illustrates the patient inclusion process.

### Rapid influenza test

All rapid influenza antigen tests were performed in the hospital's laboratories using the QuickVue Influenza A+B rapid influenza antigen test (Quidel Corp., San Diego, CA, USA), according to the manufacturer's instructions.

### Reverse-transcription polymerase chain reaction

We extracted viral nucleic acids from nasopharyngeal swab specimens with the MagNA Pure LC Total Nucleic Acid Isolation Kit (Roche Diagnostics, Mannheim, Germany), using the manufacturer's external lysis protocol and extraction reagents. Influenza virus was detected using TaqMan one-step RT-PCR Master mix reagent (ABI). Influenza A/B master mix and extracted RNA were subjected to RT-PCR in the presence of influenza matrix gene-specific primers. An internal control assay was performed with RNase P master mix containing RNase P gene-specific primers. The reactions were performed and analyzed with an ABI PRISM 7000/7900HT sequence detection system (Applied Biosystems, Branchburg, NJ, USA) or Bio-Rad CFX 96 system (Bio-Rad Laboratories, Hercules, CA, USA) under the following conditions: 30 min at 48°C and 10 min at 95°C, followed by 45 cycles of 15 s at 95°C and 1 min at 60°C.

All samples showing positivity for influenza A were typed further with influenza A swine (H1N1) nucleoprotein gene real-time RT-PCR [17], . The detection limit of the influenza A/B real-time RT-PCR was 10 copies/µL.

### Data collection

Demographic data, underlying medical conditions known to be risk factors for influenza-related complications [19], clinical features at presentation, laboratory test results, radiographic findings, influenza-related complications, and treatment course of each patient were collected from their medical records.

### Statistics

Statistical analyses were performed using the SPSS software (SPSS, Inc. Chicago, IL, USA). Student's *t*-test was used to compare continuous variables and Fisher's exact test was used to compare dichotomous variables. Variables found to be significant in univariate analyses were entered into a multivariate analysis using a logistic regression model to identify independent factors associated positively with influenza virus infection. Two-tailed *p* values<0.05 were considered to indicate statistical significance.

We calculated the sensitivity, specificity, positive and negative predictive values, and likelihood ratios (LR) for positive and negative test results. Receiver operating characteristic curves were plotted for single symptoms and various symptom combinations. The diagnostic accuracy of single and combined symptoms was assessed by calculating area under the receiver operating characteristic (AUROC) curves.

## Results

Among the 226 patients with 2009 influenza A virus (H1N1) infection, the most common symptoms were fever and respiratory symptoms. Gastrointestinal symptoms, including nausea/vomiting, diarrhea, and abdominal pain, were observed in 36% of pediatric patients and 17% of adult patients.

### Children

Demographic information and underlying comorbid conditions of the 111 children in the study sample are presented in Table 1. Most (83%) children were older than 5 years of age. The mean age of children with 2009 influenza A virus (H1N1) infection was greater than that of children with non-influenza illnesses (*p*<0.001). ILI was identified in 76% (84/111) of children with 2009 influenza A virus (H1N1) infection. Children with 2009 influenza A virus (H1N1) infection were less likely to have underlying medical conditions known to be risk factors for severe influenza [21] than were children with non-influenza illnesses.

**Table 1:** Demographic characteristics and comorbidities in patients with non-influenza illness and 2009 influenza A virus (H1N1) infection.

Characteristics	Children			Adults		
	Non-influenza (*n* = 117)*n* (%)	2009 Influenza A virus (H1N1) (*n* = 111) n (%)	*p*	Non-influenza (*n* = 202)*n* (%)	2009 Influenza A virus (H1N1) (*n* = 115) n (%)	*p*
Age (years)						
Mean (range)	5.1 (0.02–17.3)	10.2 (1.1–17.8)	<0.001	42.2 (19.3–97.1)	30.5 (18.1–83.5)	<0.001
<5	75 (64)	19 (17)				
≥65			<0.001	29 (14)	2 (2)	<0.001
Male gender	64 (55)	77 (69)	0.023	72 (36)	53 (46)	0.067
Comorbid conditions	40 (34)	27 (24)	0.145	88 (44)	24 (21)	0.000
Asthma/COPD	21 (18)	21 (19)	0.825	19 (9)	7 (6)	0.300
Cardiac/cardiovascular disease	3 (3)	1 (1)	0.622	18 (9)	7 (6)	0.370
Neurological impairment	9 (8)	4 (4)	0.281			
Diabetes mellitus	0	1 (1)	0.485	24 (12)	6 (5)	0.051
Hypertension				36 (18)	7 (6)	0.003
Dyslipidemia				12 (6)	2 (2)	0.080
Immunocompromised	1 (1)	0	1	23 (11)	3 (3)	0.006
Hospitalization		53 (49)			11 (10)	

COPD, chronic obstructive pulmonary disease.

Among the 111 children with 2009 influenza A virus (H1N1) infection, 53 (48%) were admitted to the hospital. The mean length of hospital stay was 4.3 d. Eighteen (34%, 18/53) children had at least one underlying condition. Hospitalized children were significantly younger (mean age: 8.5 vs. 12.1 years) and more likely to have underlying conditions (*p* = 0.034) than those who were not hospitalized. Eight children (7.2%, 8/111) had influenza-associated complications. One child with mosaic monosomy 14 and epilepsy developed frequent seizures, which resulted in rhabdomyolysis and required admission to the intensive care unit. Six previously healthy children developed influenza-associated pneumonia. A previously healthy 4-year-old female developed acute myocarditis and died 2 days later.

Laboratory tests were performed on specimens collected from 65 (59%, 65/111) children. Eighteen children had leukocyte counts <5000/µL and only three had leukocyte counts >15000/µL. Children with 2009 influenza A virus (H1N1) infection were more likely to have leucopenia than were children with non-influenza illness (*p* = 0.015). Antiviral therapy with oseltamivir was administered to 84 (76%, 84/111) children, the youngest of whom was 1.1 years of age. Thirty-eight (34%, 38/111) children received parenteral or oral antibacterial therapy. Children with 2009 influenza A virus (H1N1) infection were more likely to receive oseltamivir or antibiotic treatment than were children with non-influenza illness (*p*<0.001).

Univariate analysis (Table 2) showed that children with 2009 influenza A virus (H1N1) infection were more likely than those who did not have influenza to have cough (*p* = 0.001), sore throat (*p*<0.001), headache (*p*<0.001), malaise (*p*<0.001), myalgia (*p*<0.001), and ILI (*p*<0.001). Multivariate analysis showed that sore throat and ILI significantly increased the odds of having 2009 influenza A virus (H1N1) infection by more than 3-fold (OR = 3.9, 95% CI: 1.9–7.3) and 7-fold (OR = 7.5, 95% CI: 4.00–14.2), respectively.

**Table 2:** Presenting symptoms in patients with non-influenza illness and 2009 influenza A virus (H1N1) infection.

Symptoms		Children				Adults			
		Non-influenza(*n* = 117)*n* (%)	2009InfluenzaA (H1N1)(*n* = 111)*n* (%)	Univariate analysis*p*	Multivariate analysis*p*	Non-influenza(*n* = 202)*n* (%)	2009InfluenzaA (H1N1)(*n* = 115)*n* (%)	Univariate analysis*p*	Multivariate analysis*p*
Fever		110 (94)	110 (99)	0.066		156 (77)	101 (88)	0.021	
Respiratory symptoms	Cough	94 (80)	105 (95)	0.001		109 (54)	98 (85)	<0.001	<0.001
	Coryza	74 (63)	79 (71)	0.203		66 (33)	38 (33)	0.970	
	Sore throat	21 (18)	64 (58)	<0.001	<0.001	93 (46)	75 (65)	0.001	
	Any	102 (87)	109 (98)	0.002		153 (76)	110 (96)	<0.001	
Constitutional symptoms	Headache	18 (15)	48 (43)	<0.001		38 (19)	26 (23)	0.418	
	Malaise	7 (6)	26 (23)	<0.001		40 (20)	35 (30)	0.032	
	Myalgia	24 (21)	65 (59)	<0.001		96 (48)	70 (61)	0.025	
	Any	36 (31)	86 (77)	<0.001		120 (59)	95 (85)	<0.001	<0.001
Gastrointestinal symptoms	Nausea/vomiting	34 (29)	26 (23)	0.334		18 (9)	14 (12)	0.354	
	Abdominal pain	16 (14)	14 (13)	0.812		12 (6)	0	0.005	
	Diarrhea	24 (21)	13 (12)	0.072		22 (11)	9 (8)	0.377	
	Any	54 (46)	40 (36)	0.121		41 (20)	19 (17)	0.409	
Influenza-like illness		27 (23)	84 (76)	<0.001	<0.001	70 (35)	83 (72)	<0.001	

We further evaluated the performance of individual symptoms and symptom combinations as well as the rapid diagnostic test for the diagnosis of influenza infection (Table 3). ILI showed a sensitivity of 78% and a specificity of 77% in differentiating 2009 influenza A virus (H1N1) infection from non-influenza illness. No single symptom or a combination yielded a positive LR>10 (data were not shown). The specificity and sensitivity of the rapid influenza diagnostic test (RIDT) was 100% and 57%, respectively. The sensitivity of the rapid influenza diagnostic test was lower than any individual clinical symptom or a combination except for headache and malaise alone. Considering relative insensitivity of the rapid influenza diagnostic test, we further evaluated the performance of individual symptoms and symptom combinations in the diagnosis of influenza virus infection for those with a negative result of RIDT but a positive result of RT-PCR. Table 4 lists the LRs for various combinations of symptoms in pediatric patients. There was no statistical significance in the value of AUROC between a combination of cough and any constitutional symptom and ILI (*p* = 0.29). As well, no single symptom or any combination yielded a positive LR>10. The negative LR of the combination of fever and cough was 0.096 (95% CI: 0.01–0.69), which was lower than those of other symptoms and combinations, including ILI.

**Table 3:** Sensitivity and specificity of symptom and sign combinations for the identification of 2009 influenza A virus (H1N1) infection.

Symptoms and signs		non-influenza illness *vs.*2009 influenza A virus (H1N1) infection			
		children		adults	
		Sensitivity (%)	Specificity (%)	Sensitivity (%)	Specificity (%)
Single symptom or sign	Fever	99	6	88	23
	Cough	95	20	85	46
	Coryza	71	34	33	67
	Sore throat	58	82	65	54
	Any respiratory symptom	98	13	96	24
	Headache	43	85	23	81
	Malaise	23	94	30	80
	Myalgia	59	80	61	52
	Any constitutional symptom	78	69	83	40
Symptom and sign combinations	Fever + cough	94	23	74	59
	Any respiratory + any constitutional symptom	77	74	79	56
	Fever + any respiratory symptom	97	17	84	43
	Fever + any constitutional symptom	77	73	76	53
	Cough + any constitutional symptom	74	78	70	71
	Influenza-like illness	76	77	72	65
Rapid influenza diagnostic test		57	100	38	100

**Table 4:** Sensitivity and specificity of symptom and sign combinations for the identification of 2009 influenza A virus (H1N1) infection in patients with negative rapid influenza diagnostic tests (RIDTs).

Symptoms and signs		Non-influenza illness with RIDT (−) and RT-PCR (−) *vs.* H1N1 with RIDT (−) and RT-PCR (+)					
		Children			Adults		
		AUROC (%)	Positive LR(95% CI)	Negative LR(95% CI)	AUROC (%)	Positive LR(95% CI)	Negative LR(95% CI)
Single symptom or sign	Fever	51.9	1.04 (0.98–1.11)	0.37 (0.05–2.93)	52.6	1.07 (0.93–1.22)	0.78 (0.44–1.37)
	Cough	59.8	1.25 (1.14–1.36)		69.3	1.71 (1.49–1.98)	0.16 (0.07–0.38)
	Coryza	50.6	1.02 (0.79–1.32)	0.97 (0.61–1.53)	53.9	1.12 (0.94–1.32)	0.76 (0.48–1.20)
	Sore throat	71.0	3.34 (2.12–5.26)	0.49 (0.34–0.70)	62	1.52 (1.23–1.88)	0.55 (0.37–0.81)
	Any respiratory symptom	56.4	1.15 (1.07–1.23)		61.9	1.31 (1.22–1.42)	
	Headache	63.4	2.75 (1.59–4.74)	0.68 (0.53–0.89)	50.6	1.01 (0.89–1.15)	0.94 (0.52–1.69)
	Malaise	62.6	5.21 (2.25–12.0)	0.73 (0.60–0.90)	58.5	1.86 (1.22–2.82)	0.79 (0.65–0.96)
	Myalgia	69.7	2.92 (1.90–4.48)	0.50 (0.35–0.73)	54.1	1.17 (0.91–1.51)	0.84 (0.63–1.14)
	Any constitutional symptom	74.6	2.60 (1.91–3.55)	0.29 (0.16–0.52)	60.6	1.36 (1.15–1.59)	0.47 (0.28–0.80)
Symptom and sign combinations	Fever + cough	60.4	1.27 (1.14–1.42)	0.096 (0.01–0.69)	67.2	1.85 (1.49–2.29)	0.42 (0.27–0.65)
	Any respiratory + any constitutional symptom	77.2	3.12 (2.22–4.39)	0.27 (0.15–0.49)	67.2	1.82 (1.50–2.20)	0.34 (0.21–0.57)
	Fever + any respiratory symptom	57.4	1.18 (1.07–1.30)	0.13 (0.02–0.94)	62.6	1.44 (1.22–1.69)	0.41 (0.24–0.71)
	Fever + any constitutional symptom	75.2	2.84 (2.04–3.97)	0.31 (0.18–0.53)	61	1.47 (1.18–1.82)	0.58 (0.40–0.85)
	Cough + any constitutional symptom	78.9	3.60 (2.49–5.21)	0.26 (0.14–0.46)	73.1	2.61 (2.02–3.38)	0.35 (0.23–0.53)
	Influenza-like illness	77.3	3.37 (2.34–4.85)	0.29 (0.17–0.50)	67.1	1.98 (1.55–2.54)	0.47 (0.33–0.69)

RIDT, rapid influenza diagnostic test; RT-PCR, reverse transcriptase polymerase chain reaction; AUROC, area under the receiver operating characteristic curve; LR, likelihood ratio.

### Adults

Demographic information and underlying comorbid conditions of the 115 adult patients are presented in Table 1. Only two patients were more than 65 years of age. The mean age of individuals with 2009 influenza A virus (H1N1) infection was lower than that of patients with non-influenza illness (*p*<0.001). ILI was identified in 72% (83/115) of the adult patients with 2009 influenza A virus (H1N1) infection. Adult patients with 2009 influenza A virus (H1N1) infection were less likely than those with non-influenza illness to have underlying medical conditions known to be risk factors for severe influenza (*p*<0.001) [21].

Eleven (10%, 11/115) of the adult patients with 2009 influenza A virus (H1N1) infection were admitted to the hospital, eight (73%, 8/11) of whom had at least one underlying condition. The mean length of hospital stay was 5.7 d, excepting one patient who was transferred to a nursing home. Hospitalized patients were significantly older (mean age: 52.6 vs. 28.1 years) and more likely to have underlying conditions (*p*<0.001) than those who were not hospitalized. Seven (6%, 7/115) patients had influenza-associated complications; all had pneumonia. Five (71%, 5/7) of these patients had other underlying medical conditions. Two developed respiratory failure, requiring admission to the intensive care unit and mechanical ventilation. No death occurred.

Laboratory test results were available for 24 (21%, 24/115) patients. Only two cases had leukocyte counts <5000/µL and none had a leukocyte count >15000/µL. There was no significant difference in the incidence rate of leucopenia between adult patients with 2009 influenza A virus (H1N1) infection and those with non-influenza illness (*p* = 0.732). Antiviral therapy with oseltamivir was administered to 66 (57%, 66/115) patients. Nineteen (17%, 19/115) patients received parenteral or oral antibacterial therapy, with or without oseltamivir treatment. Similar to the results in children, adult patients with 2009 H1N1 infection were more likely than those with non-influenza illness to receive oseltamivir or antibiotic treatment (*p*<0.001).

Univariate analysis (Table 2) showed that adults with 2009 influenza A virus (H1N1) infection were more likely than those who did not have influenza to have fever (*p* = 0.021), cough (*p*<0.001), sore throat (*p* = 0.001), malaise (*p* = 0.032), myalgia (*p* = 0.025), and ILI (*p*<0.001), and less likely to have abdominal pain (*p* = 0.005). Multivariate analysis showed that cough and constitutional symptoms (headache, malaise, myalgia) increased the odds of having 2009 influenza A virus (H1N1) infection by more than 5-fold (OR = 5.6, 95% CI: 3.1–10.2) and 3-fold (OR = 3.7, 95% CI: 2.1–6.7), respectively.

We further evaluated the performance of individual symptoms and symptom combinations as well as the rapid diagnostic test for the diagnosis of influenza infection (Table 3). ILI showed a sensitivity of 69% and a specificity of 65% in differentiating 2009 influenza A virus (H1N1) infection from non-influenza illness. As seen in the children, no single symptom or any combination yielded a positive LR>10 (data were not shown). The specificity and sensitivity of the rapid influenza diagnostic test in the diagnosis of influenza was 100% and 38%, respectively. The sensitivity of the rapid influenza diagnostic test was lower than any individual clinical symptom or a combination except for coryza, headache and malaise. Table 4 lists the LRs for various combinations of symptoms associated with 2009 influenza A virus (H1N1) infection in adult patients with negative results of RIDTs. A combination of cough and any constitutional symptom yielded an AUROC of 73.1%, which was larger than other symptom or combination, including ILI (67.1%, *p*<0.05). No single symptom or any combination yielded a positive LR>10 or a negative LR<0.1.

## Discussion

Acute respiratory illnesses are the leading cause of outpatient medical visits among patients of all ages. Although it is neither necessary nor cost-effective to establish a specific viral cause for most respiratory viral diseases, it is important to distinguish influenza from other respiratory viruses because the influenza virus is associated with higher morbidity and mortality and early antiviral treatment can reduce the risk of severe illness or death [20]–[26]. In addition, the rapid detection of influenza viruses also can prompt strategies to prevent transmission to other patients. Among the laboratory-based methods of influenza diagnosis, rapid influenza diagnostic tests have been adopted increasingly by clinicians because they provide test results within 15 min.

Symptomatic predictors of influenza have been examined using surveys and clinical trials and in practice settings [27], [28]. The sensitivity of clinical predictors for influenza varies depending on a multitude of factors, including the prevalence of disease, age, underlying illnesses, duration of symptoms prior to consultation, and the vaccination rate in the population tested. Thus, the use of symptomatic predictors should be limited to periods of known influenza virus circulation [3], [4], [28], [29]. Most studies, including ours, have been conducted in the context of community outbreaks of seasonal influenza. In the present study, sore throat and ILI increased the probability of 2009 influenza A virus (H1N1) infection in pediatric patients. Among adults, cough and constitutional symptoms increased the probability of 2009 influenza A virus (H1N1) infection.

Although previous studies have shown that fever and cough were better predictors of influenza virus infection, no symptom or combination has been found to be sufficiently specific for the diagnosis of influenza virus infection [3], [4], [28], [29]. This was also true in the present study. A wide range of sensitivities of the rapid influenza tests have been reported, whereas specificities have been reported to be high [6], [9]. In this study, we found that the rapid influenza diagnostic test had relatively low sensitivity (38–57%) but excellent specificity (100%) for the detection of 2009 influenza A virus (H1N1) infection, consistent with the study by Uyeki et al [30]. Compared with any symptoms or combinations, the sensitivity of the rapid influenza diagnostic test was lower. Because of the lower sensitivity and moderate negative predictive value (71–74%) of rapid influenza diagnostic tests, we undertook a further evaluation to identify symptomatic predictors for patients with negative results of RIDT. We found that a combination of cough and any constitutional symptom was more accurate than other symptoms or combinations in predicting 2009 influenza A virus (H1N1) infection in children and adults with negative RIDT results. However, the positive predictive values were only 58% and 47%, respectively. In contrast, we found that the combination of fever and cough had a sufficiently negative LR (0.096) to exclude the probability of 2009 influenza A virus (H1N1) infection in pediatric patients with negative RIDT results, and had a negative predictive value (NPV) of 87.6% in adult patients. These results indicate that without fever and cough, the probability of influenza virus infection in patients with negative RIDT results, was low, to a level of 10% or so. The sensitivity and predictive values of ILI criteria for the diagnosis of influenza have varied among studies, with positive predictive values ranging from 23% to 81% [28], [31]. In the present study, a combination of cough and any constitutional symptom had a better sensitivity and positive predictive value than the ILI criteria for the diagnosis of influenza in patients with negative RIDT results. Further prospective studies may be needed to validate our findings.

For a retrospective study in nature, there are intrinsic limitations in the current study. First, all the symptoms and signs were collected from the medical charts, which were recorded by the physicians but not by a designed checklist, so some symptoms and signs may be missed. Second, the timings of samplings for diagnostic tests were inconsistent, which may affect the accuracy rate of diagnostic tests and the subsequent analyses. Third, for the homogeneity of patients, we only studied the patients with 2009 influenza A virus (H1N1) infection, so whether the results presented here can be applied to other subtypes of influenza virus infection needs further observations. Forth, the study was conducted during the epidemic of influenza, so it should be more cautious when applying these results during non-epidemic. In contrast, we not only evaluated the patients with influenza virus infection, but also the influenza-infected patients with a negative rapid diagnostic test, which provides important information for the clinicians in daily clinical practice.

### Conclusions

Clinical symptoms alone are inadequate to confirm the diagnosis of influenza. Clinicians must pay attention to survey data to recognize whether influenza viruses are circulating. In influenza epidemic settings, clinicians should be aware that rapid influenza diagnostic tests are relatively insensitive for the diagnosis of influenza virus infection. For patients with negative rapid influenza diagnostic results, those lacking fever and cough have a low probability of influenza virus infection. The best management strategy should be made on a case-by-case basis and depend on the severity of illness.
